# 30 day predicted outcome in undifferentiated chest pain: multicenter validation of the HEART score in Tunisian population

**DOI:** 10.1186/s12872-021-02381-z

**Published:** 2021-11-19

**Authors:** Mohamed Hassene Khalil, Adel Sekma, Hajer Yaakoubi, Khaoula Bel Haj Ali, Mohamed Amine Msolli, Kaouthar Beltaief, Mohamed Habib Grissa, Hamdi Boubaker, Mohamed Sassi, Hamadi Chouchene, Youssef Hassen, Houda Ben Soltane, Zied Mezgar, Riadh Boukef, Wahid Bouida, Semir Nouira

**Affiliations:** 1 Emergency Department and Laboratory Research (LR12SP18), Fattouma Bourguiba University Hospital, 5000 Monastir, Tunisia; 2 Emergency Department, Sahloul University Hospital, 4011 Sousse, Tunisia; 3 Research Laboratory LR12SP18, University of Monastir, 5019 Monastir, Tunisia; 4 Emergency Department, Farhat Hached University Hospital, 4031 Sousse, Tunisia

**Keywords:** Chest pain, Severity of illness index, Myocardial infarction, Admission avoidance, Emergency service

## Abstract

**Background:**

Chest pain remains one of the most challenging serious complaints in the emergency department (ED). A prompt and accurate risk stratification tool for chest pain patients is paramount to help physcian effectively progrnosticate outcomes. HEART score is considered one of the best scores for chest pain risk stratification. However, most validation studies of HEART score were not performed in populations different from those included in the original one.

**Objective:**

To validate HEART score as a prognostication tool, among Tunisian ED patients with undifferentiated chest pain.

**Methods:**

Our prospective, multicenter study enrolled adult patients presenting with chest pain at chest pain units. Patients over 30 years of age with a primary complaint of chest pain were enrolled. HEART score was calculated for every patient. The primary outcome was major cardiovascular events (MACE) occurrence, including all-cause mortality, non-fatal myocardial infarction (MI), and coronary revascularisation over 30 days following the ED visit. The discriminative power of HEART score was evaluated by the area under the ROC curve. A calibration analysis of the HEART score in this population was performed using Hosmer–Lemeshow goodness of test.

**Results:**

We enrolled 3880 patients (age 56.3; 59.5% males). The application of HEART score showed that most patients were in intermediate risk category (55.3%). Within 30 days of ED visit, MACE were reported in 628 (16.2%) patients, with an incidence of 1.2% in the low risk group, 10.8% in the intermediate risk group and 62.4% in the high risk group. The area under receiver operating characteristic curve was 0.87 (95% CI 0.85–0.88). HEART score was not well calibrated (χ^*2*^ statistic = 12.34; p = 0.03).

**Conclusion:**

HEART score showed a good discrimination performance in predicting MACE occurrence at 30 days for Tunisian patients with undifferentiated acute chest pain. Heart score was not well calibrated in our population.

## Background

Chest pain remains one of the most common, potentially serious presenting complaints for adults emergency department [1] visits with approximately 7.6 million yearly visits in the United States [2]. The priority for emergency physician is to determine whether these patients with acute chest pain have a potential life threatening underlying etiology. The great challenge is to differentiate patients presenting with acute coronary syndrome [3] and those with other more benign conditions. Obviously, medical history, clinical examination, and laboratory values may help to identify patients with true ACS. None is sufficiently accurate to be used independently [4]. Thus, more than 2% of ACS patients are inappropriately discharged annually [5]. Therefore, there is a global tendency for ED physician to overinvestigate chest pain patients with further, often more invasive testing, even in low risk patients. This kind of practice leads to resource overutilization and a huge health costs waste contrasting with no outcomes improvement [6].

For many years, physicians were searching tools, ranging from specific diagnostic tests to entire strategies of evaluation, to appropriately risk stratify patients with chest pain in order to simultaneously prevent major adverse cardiac events [3] and reduce unnecessary testing and hospitalisations. Based on the principal that a prompt quick and accurate identification of patients who are at high and low risk of developing major adverse cardiac events is paramount, and in order to optimally allocate ED and hospital resources, many bioclinical scores have been developed. HEART score is one of the more recently proposed model derived through a process involving expert opinion and review of medical literature. It is calculated based on admission data of medical history, EKG, age, cardiovascular risk factors and troponin levels [7]. The HEART score was created specifically to identify ED patients presenting with undifferentiated chest pain who were at low risk as well as patients at high risk of short-term MACE occurrence. Several scientific societies are encouraging the use of HEART score, for evaluating patients with chest pain suggestive of ACS in the ED [8, 9]. However, most of validating HEART score studies targeted Caucasian populations in high-income countries with scarce validation in other ethnic groups. For this reason, HEART score needs to be tested in various populations to check its accuracy and the eventual need for customization [10, 11]. Additional studies providing further worldwide data about the validation of this risk score will empower emergency physicians’ decision making when relying on this score in ruling in or ruling out their chest pain patients. The goal of our investigation is to validate HEART score as a prognostication tool among ED patients with chest pain in teaching hospitals in Tunisia.

## Methods

### Study design and setting

We conducted a cohort study of ED patients who were admitted to chest pain units in the ED of 3 Tunisian hospitals: Fattouma Bourguiba University Hospital, Monastir; Sahloul University Hospital, Sousse and Farhat Hached University Hospital, Sousse.

### Ethics statement

The study’s objectives and procedures were approved by the local independent ethics committee. It is conducted in accordance with the principles of the Declaration of Helsinki. All patients and/or their surrogates received written information about the study and provided their verbal consent to participate. The present manuscript was drafted in compliance with the STROBE checklist for cohort studies.

### Selection of participants

Patients with a chief complaint of chest pain were prospectively recruited during the period from January 2015 to April 2017. Our inclusion criteria comprised any adult aged older than 30 years with a chief complaint of ‘‘chest pain’’, ‘‘chest tightness’’, or ‘‘chest pressure’’. We excluded subjects with an ST segment elevation myocardial infarction (STEMI) and patients lost to follow up. We also excluded patients in whom an obvious diagnosis was made immediately after initial medical evaluation.

### HEART score measurement

HEART score calculation relies on five elements [12]: history, EKG, age, risk factors, and troponin. The history component was scored using a list of predefined chest pain characteristics that were categorized as typical or atypical. Chest pain was considered as typical chest discomfort if it is characterized by a retrosternal or precordial sensation of pain, pressure, or tightness radiating to the left arm, the neck, or the jaw, either intermittent or persistent, occurring at rest or related to exercise. Chest pain was considered as atypical when patient related different chest location of the pain, or different radiation than described below. The history was classified as «non-suspicious» if it contained only atypical characteristics. If the patient history contained both typical and atypical characteristics, the history was classified as «moderately suspicious». If the history contained only typical elements, the history was classified highly suspicious.

The EKG component was scored based on the impression of the first EKG by the treating physician. The measured troponin values were interpreted according to local lab standards and reference values. Troponin T (Roche, Diagnostics (Basel, Switzerland), lower limit of detection 0.01 ng/mL; 99th centile < 0.01 ng/mL; 10% coefficient of variation 0.035 ng/mL) was used during the study period. Troponin T > 0.01 was considered positive. Only the troponin value of the first blood sample was used for the HEART score calculation. High sensitive troponin was not used at the time of the study conduct. The sum of points given for each component defined the HEART score value. The standard version of the HEART score was used [7]. Patients with a HEART score of 0 to 3 were categorized as low risk to develop MACE. Patients with a HEART score between 4 and 6 were categorized as intermediate risk and a HEART score of 7 or upper was considered at high risk of MACE occurrence.

### Outcome measures

The primary outcome was MACE occurrence including all-cause mortality, non-fatal myocardial infarction (MI), and coronary revascularisation within 30 days. The diagnosis of MI was defined according to the third definition of myocardial infarction [13]. All study files were reviewed by two independent, experienced physicians, a cardiologist and an emergency physician. When disagreement occurs, a third opinion was asked from another experienced cardiologist. To check for MACE occurrence at day 30, data were collected through direct phone calls. If required, patients were reconvened to check their health records to ensure that myocardial infarction or coronary intervention occurred during the one-month period after discharge.

### Statistical analysis

We aimed to find a difference in one month MACE between low, intermediate and high-risk categories of HEART score. The power calculation for our study assumes an alpha of 0.05 and beta of 0.1 looking for a false negative rate of 1% in the low risk group yielding a sample size of at least 3874 patients. Since we anticipated that 5–10% of patients would be excluded, we aimed for a sample size of 4260. All continuous data are presented as either the median with the interquartile range or the mean with Standard Deviation (SD), according to the distribution of the data. The categorical data are presented as the percentage of occurrence. We used Student’s t test for comparison of means for continuous variables, Chi-square and Fisher’s exact test for comparison of categorical variables. We calculated the area under the receiver operating characteristic curve (ROC) with its binomial exact 95% confidence interval for the HEART score in predicting the occurrence of MACE within one month of ED visit. Calibration analysis to assess whether the observed MACE rate match expected MACE rate was tested using the Hosmer–Lemeshow goodness of fit test. Statistical significance was defined as *p* < 0.05, two-sided. Statistical analysis was carried out using IBM SPSS Statistics for Windows software version 20.0^®^.

## Results

**Fig. 1:**
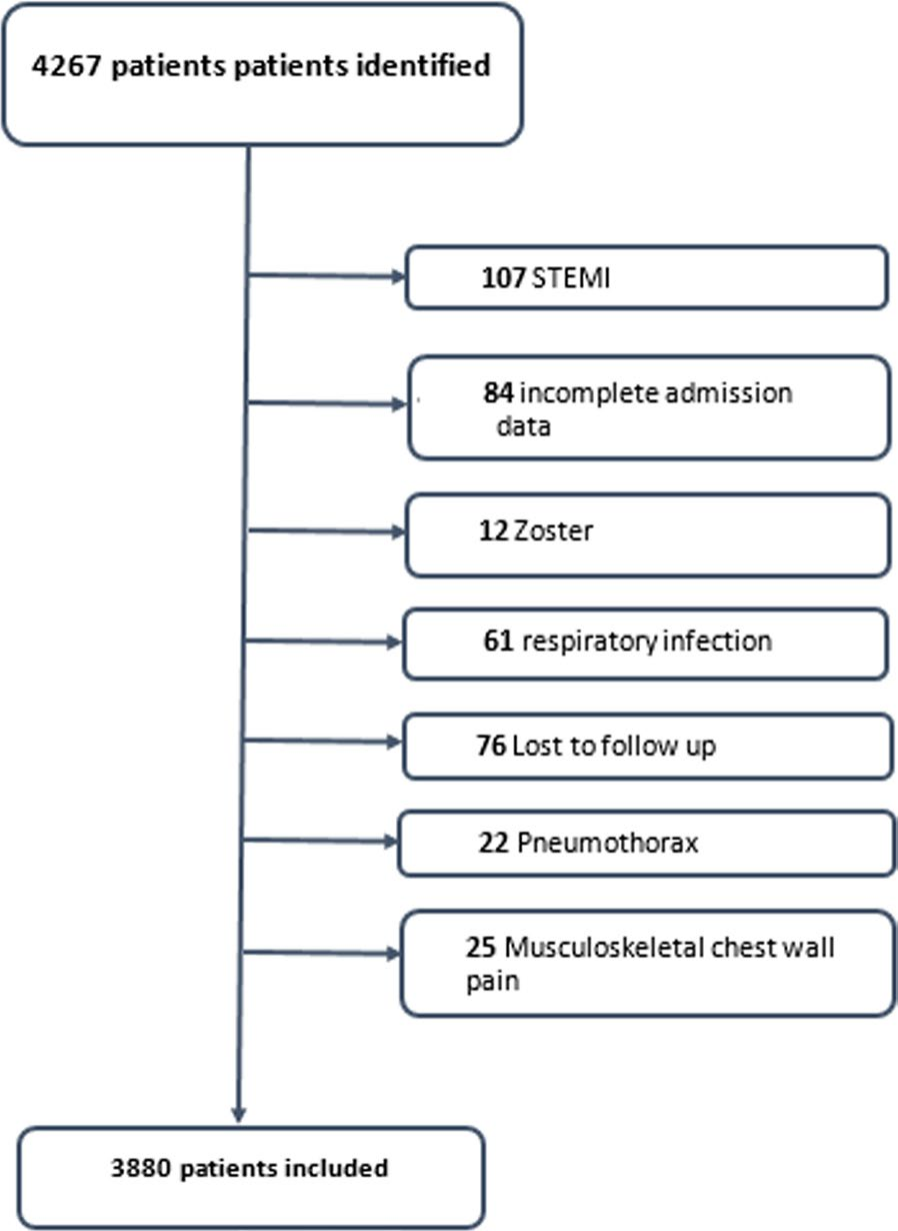
Flowchart of the inclusion process

**Fig. 2:**
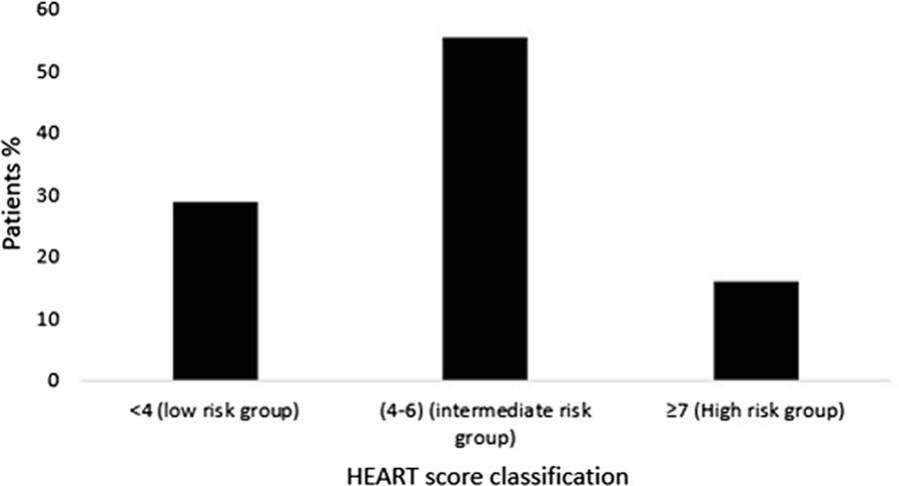
Patients’ distribution in each risk group of HEART score

**Fig. 3:**
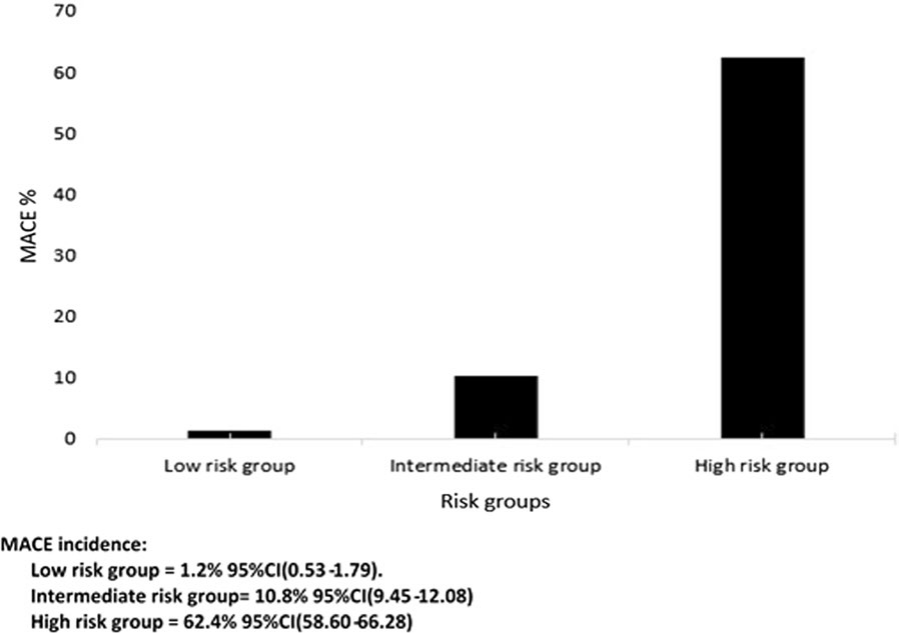
MACE rate in each HEART score Group

**Fig. 4:**
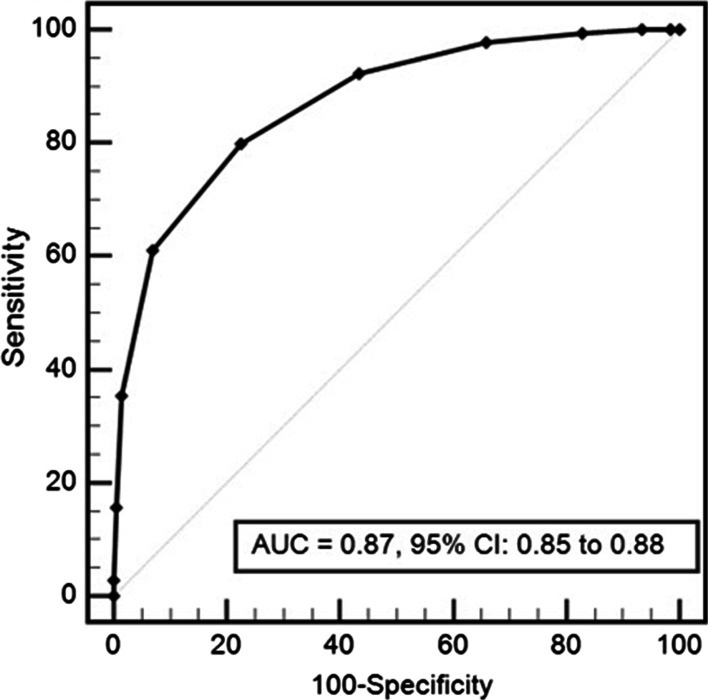
Receiver operating characteristic curve for HEART score in predicting MACE at 30 days. AUC = 0.87, 95% CI: 0.85 to 0.88

During the study period, we enrolled 4267 patients; we excluded 387 patients (Fig. 1). The main exclusion criterion was lost for follow up. This resulted in 3880 subjects included in our study. Baseline characteristics of the study population are shown in Table 1. Mean age was 56.3 years with male/female ratio of 1.47. Diabetes and hypercholesterolemia were the most prevalent cardiovascular risk factors (43% and 36.8% respectively). There were 2959 (76.3%) patients discharged home from the ED, 409 (10.5%) admitted to coronary care unit, and 512 (13.2%) were admitted to the ward. For risk stratification groups, we noticed that most of our patients were classified as intermediate risk score (55.3%) (Fig. 2). Within 30 days of ED visit, MACE were reported in 628 (16.2%) patients. Among these patients, 122 developed non-fatal myocardial infarction and 484 underwent coronary intervention: 350 (9%) underwent percutaneous coronary intervention 45 (1.2%) had a coronary artery bypass graft surgery and 89 (2.3%) had a coronary angiography with obvious stenosis but managed conservatively. The distribution of major cardiovascular events in the different risk groups at 30-day assessment is shown in Fig. 3. The numerical distribution of the score’s five components in the groups with and without MACE differed significantly (Table 2). Among the five components, ECG and troponin showed remarkably significant positive likelihood ratio for MACE occurrence. The average HEART score was 4.16 ± 1.7 in non-MACE group and 6.84 ± 1.6 in MACE group. The ability of HEART score to predict MACE occurrence in the study cohort is shown in Fig. 4. The area under receiver operating characteristic curve was 0.87 (95% CI 0.85–0.88). Hosmer–Lemeshow test showed a statistically significant difference between observed and expected MACE rates (χ^2^ = 12.34 and *p* = 0.03). Table 3 shows sensitivity, specificity, positive (PPV) and negative (NPV) predictive values of HEART score using the cutoff values usually accepted.

**Table 1:** Baseline characteristics

	Included patients N = 3880	Excluded patients N = 387	p value
Variable	Value		
Mean age	56 ± 13.8	57 ± 13.6	0.103
Male sex n (%)	2307 (59.5)	242 (62.5)	0.254
*Medical history n (%)*			
Diabetes mellitus	1668 (43)	173 (44.5)	0.519
Smokers	1264 (32.6)	116 (30.1)	0.332
Coronary artery disease	851 (22)	9 (24.8)	0.199
Hypercholesterolemia	1429 (36.8)	136 (35.1)	0.543
Hypertension	1002 (25.8)	88 (22.7)	0.199
*Data at ED admission*			
Heart rate (bpm)	81 ± 15	82 ± 16	0.844
Mean systolic blood pressure (mmHg)	140 ± 20	138 ± 9	0.285
Meandiastolicblood pressure (mmHg)	78 ± 14	77 ± 12	0.523
Normal EKG	2119 (54.6%)	226 (58.4)	0.164
*Patients issue*			
Discharged	2959 (76.3%)	201 (71.8)	0.06
Coronary care unit admission	409 (10.5%)	37 (13.2)	0.121
Admitted to the ward	512 (13.2%)	36 (9.3)	0.03

ED, Emergency department

**Table 2:** Number of patients in each elements of the HEART score

	MACE						*p* value for trend	Likelihood ratio (+)	Likelihood ratio (−)
	Yes			No					
Points	0 (%)	1 (%)	2 (%)	0 (%)	1 (%)	2 (%)			
History	71 (11.3)	110 (17.5)	447 (71.2)	968 (29.8)	818 (25.2)	1466 (45.1)	< 0.001	1.57	0.52
ECG	262 (41.7)	48 (7.6)	318 (50.6)	2998 (92.2)	179 (5.5)	75 (2.3)	< 0.001	21.9	0.50
Age	45 (7.2)	333 (53)	250 (39.8)	856 (26.3)	1621 (49.8)	775 (23.8)	< 0.001	1.67	0.79
Risk factors	39 (6.2)	224 (35.7)	365 (58.1)	663 (20.4)	1636 (50.3)	953 (29.3)	< 0.001	1.98	0.59
Troponin	57 (9.1)	317 (50.5)	254 (40.4)	605 (18.7)	2556 (78.6)	91 (2.8)	< 0.001	14.45	0.61

**Table 3:** Prognostic performance of HEART score using the cutoffs of HEART score < 4 and HEART score ≥ 7

	PPV	NPV	Sensitivity	Specificity	Yuden index
Low risk^a^	22.27	98.84	97.93	34.01	0.32
High risk^b^	62.44	92.53	61.15	92.9	0.54

PPV, Positive predictive value; NPV, negative predictive value

^a^HEART score < 4

^b^HEART score ≥ 7

### Subgroups analysis

Subgroups analysis showed that area under ROC curve did not change significantly according to gender. However, it was higher in patients under the age of 65 [0.89 (95% CI 0.87–0.90)] compared to older patients (0.83 (95% CI 0.80–0.87)) *p* = 0.004. The lowest AUC value was found among the subgroup of patients with the highest level of Troponin (0.72 (95%CI 0.66–0.78)). HEART score had similar sensitivity level in both sex groups. However, sensitivity was the lowest when the level of troponin was normal (sensitivity = 82.5%). Specificity is low in all subgroups. The NPV is high in all subgroups and reaches 100% in the subgroup of patients with troponin superior to 3 times the normal limit.

## Discussion

In this study, we analyzed the accuracy of HEART score in predicting short-term risk of major adverse cardiovascular events in a large contemporary cohort of Tunisian patients presenting to the ED for acute undifferentiated chest pain. We found that HEART score has an excellent prognostic value and could serve as an effective risk stratification tool.

It is well known that patients admitted to the ED with chest pain are at risk for several life-threatening conditions. Given the difficulties associated with accurate risk stratification of these patients, and the potential consequences associated with inappropriate discharge, clinicians often elect to admit patients even whom they believe to be at low risk of MACE [14]. As a result, the AHA/ACC guidelines have recommended that risk stratification scores should be used to aid in clinical decision-making [1], but the best one to be used is still unknown. Among the widely used risk stratification scores, we mention TIMI score and the GRACE score. However, neither TIMI nor GRACE scores were designed for ED chest pain risk stratification. The relevance of their use for MACE prediction and patient disposition in ED undifferentiated chest pain is up for debate [15–18]. Boubaker et al. have shown that both scores had low prognostic value and do not serve as an effective risk stratification tool in Tunisian chest pain population [19]. Recently, the HEART score has emerged as a reliable alternative to risk stratify patients presenting with undifferentiated chest pain in emergency departments. It was firstly introduced in Europe. It is easy to use, intuitive and includes well-established factors associated with the probability of having an ACS. It helps identifying low risk patients in whom immediate further cardiac testing can be safely forgone. It was developed and validated initially in Netherlands, then validated in American and Asian populations but has not been tested in North African populations. Ethnic validation of risk scores is paramount, since inter-ethnic differences in cardiovascular risk factors potentially influence risk score performances. Thus, extrapolation of risk stratification models to different settings cannot be anticipated [20–23]. This is the first prospective study assessing HEART score risk stratification performance in a large sample of Tunisian patients with acute chest pain. Our study population is comparable to the original study population [7] and other previous cohorts validating HEART score [3, 24, 25]. However, our patients were younger. In addition, we had higher frequency of diabetes and lower rate of hypertension in our population. Like the previous validating HEART score studies, our incidence of MACE was low in the three HEART score categories, compared to the original study population [26]. Our results suggest that HEART score can be used in risk stratifying chest pain patients. A c-statistic of 0.87 for the HEART score indicates a good to excellent ability to discriminate the short-term prognosis in ED chest pain patients. Our findings are in accordance with the results of many studies that tested and validated the clinical value of the HEART score in many countries. Matthew et al. [27] reported that the AUROC of HEART score was as good as 0.88, 95% CI (0.84–0.93); they showed that there was no MACE in their low risk group patients. In a stepped-wedge, cluster randomized trial; Poldervaart et al. [28] also reported that HEART score was an accurate risk stratification instrument and safe to use when assessing patients with chest pain in the ED. In our low risk group patients, incidence of MACE was 1.2%. This finding is quite similar to that shown in the meta-analysis of Berg et al. [29]. They showed that the pooled incidence of “missed” MACE was 1.6% in the low risk group. It is noteworthy that none of our low risk patients died during the 30-day follow up. Even though, we should acknowledge that to safely rule-out ACS, the HEART score should achieve higher sensitivity and NPV [30]; its poor calibration must still urge to more caution in its use in clinical practice.

There are several potential limitations to our study that should be acknowledged. First, although our study has the largest sample size, it may not reflect the full spectrum of patients with acute non traumatic chest pain because we did not include those admitted to nonteaching hospitals or patients treated in an ambulatory setting. However, many of these patients were secondarily transferred to our emergency departments to be investigated in our chest pain units. Second, the relative short duration of follow-up in current study could not allow us to observe more clinical events. Future study is warranted to evaluate whether the HEART score is also useful for longterm risk prediction in our population. Third, the HEART score utilized conventional troponins as cardiac biomarker component; the question whether using highly sensitive troponins would improve the performance of the score is a relevant issue requiring a specific study. Fourth, we need to know more about the real effects of using the HEART score in routine clinical practice. Such experience should be investigated to provide the evidence for its potential benefit. Fifth, there were 76 (1.8%) patients lost to follow. Despite that such rate is considered low, assuming that these patients reached endpoint, there could be an impact on the estimated prognostic accuracy.

## Conclusion

Our study indicates that the HEART sore had a good performance to evaluate the risk of MACE within 30 days in Tunisian patients with undifferentiated acute chest pain. However, its use in clinical decision making should be prudent.

## Data Availability

All orignal data used to support the findigs.
